# Refractory chronic cluster headache: a consensus statement on clinical definition from the European Headache Federation

**DOI:** 10.1186/1129-2377-15-79

**Published:** 2014-11-27

**Authors:** Dimos D Mitsikostas, Lars Edvinsson, Rigmor H Jensen, Zaza Katsarava, Christian Lampl, Andrea Negro, Vera Osipova, Koen Paemeleire, Aksel Siva, Dominique Valade, Paolo Martelletti

**Affiliations:** 1 Athens Naval Hospital, Neurology Department, 77A Vas. Sofias Avenue, 11521 Athens, Greece; 2 Department of Medicine, Institute of Clinical Sciences, Lund University, Lund, Sweden; 3 Danish Headache Center, Department of Neurology, University of Copenhagen, Glostrup Hospital, Copenhagen, Denmark; 4 Department of Neurology, Evangelical Hospital, Unna, and Department of Neurology, University of Duisburg-Essen, Essen, Germany; 5 Headache Center Seilerstaette, Department of Neurogeriatric medicine and Remobilisiation, Hospital Barmherzige Schwestern Linz, Linz, Austria; 6 Department of Clinical and Molecular Medicine, Sapienza University of Rome, Rome, Italy; 7 First Sechenov Moscow State Medical University, Moscow, Russia; 8 Department of Neurology, Ghent University Hospital, Ghent, Belgium; 9 Neurology Department, Istanbul University Cerrahpaşa School of Medicine, Istanbul, Turkey; 10 Emergency Headache Centre, Lariboisière Hospital, Paris, France; 11 Regional Referral Headache Centre, Sant’Andrea Hospital, Rome, Italy

**Keywords:** Chronic cluster headache, Refractory chronic cluster headache, Invasive treatments

## Abstract

Chronic cluster headache (CCH) often resists to prophylactic pharmaceutical treatments resulting in patients’ life damage. In this rare but pragmatic situation escalation to invasive management is needed but framing criteria are lacking. We aimed to reach a consensus for refractory CCH definition for clinical and research use. The preparation of the final consensus followed three stages. Internal between authors, a larger between all European Headache Federation members and finally an international one among all investigators that have published clinical studies on cluster headache the last five years. Eighty-five investigators reached by email. Proposed criteria were in the format of the International Classification of Headache Disorders III-beta (description, criteria, notes, comments and references). Following this evaluation eight drafts were prepared before the final. Twenty-four (28.2%) international investigators commented during two rounds. Refractory CCH is described in the present consensus as a situation that fulfills the criteria of ICHD-3 beta for CCH with at least three severe attacks per week despite at least three consecutive trials of adequate preventive treatments. The condition is rare, but difficult to manage and invasive treatments may be needed. The consensus addresses five specific clinical and paraclinical diagnostic criteria followed by three notes and specific comments. Although refractory CCH may be not a separate identity these specific diagnostic criteria should help clinicians and investigators to improve patient’s quality of life.

## Introduction

Cluster headache (CH) is a rare but severe primary headache with a circadian and circannual pattern, characterized by periorbital unilateral pain and untreated headache attacks shorter than 3 hours accompanied by ipsilateral autonomic symptoms. It belongs into the Trigeminal Autonomic Cephalalgias (TACs) category of Primary Headache Disorders [1]. Depending on the attack frequency CH is classified into episodic (ECH) and chronic CH (CCH). About one person every 1,000 adults experiences CH. Males are affected four times more often than females overall, but this difference increases up to 15 holds in the case of chronic CH. Episodic CH is six times more common than CCH. The prevalence seems to be stable among adult ages and countries [2]. For a proportion of patients a genetic predisposition has been hypothesized as 2% to 7% of patients have one or more affected relatives [3]. The evolution from an episodic form can require years but CH can also be chronic since its onset. It has been observed that one out of three patients with ECH at onset will develop CCH 10 years later. On the other hand, the same proportion of patients with CCH at onset turns into episodic within 10 years later while nearly the half with CCH at onset still has CCH after 20 or more years [4]. Because of its severity CH has a large socioeconomic impact and associated morbidity; almost 80% of patients report restricting daily activities [5].

Among headache sufferers seeking neurological consultation in Europe only 3% suffer from CH, though in primary care settings this percentage falls to 1% [6], indicating that CH is largely misdiagnosed [7,8]. The socio-economic burden of CH on the individual and society is because of the direct costs of healthcare services, and the indirect costs of lost workdays and decreased work efficacy. In Denmark has been estimated that less than 50% of CH patients are treated by headache specialists, approximately 30% had missed work and 78% report restrictions in daily living [9]. In Germany the annual cost of a single CCH patient is over €21,000 [10]. Yet the personal burden is enormous for those patients who do not respond to treatment, since CH is a highly disabling condition with pain that ranks among the most severe known to humans [11,12].

### Why do we need criteria to define refractory chronic Cluster Headache?

1. Severity of attacks varies largely in CCH patients.

According to the ICHD-3 beta CCH is defined as a condition with attacks occurring without a remission period, or with remissions lasting <1 month, for at least 1 year [1]. This definition does not considerate the wide range of severity of the condition, as a patient could have three attacks a month or three attacks a day, in both cases for a year or more. A further definition that takes into account the disability caused by CCH may be needed.

2. Available treatments are not always efficient leaving patients without pain remission.

Treatments with a good clinical experience for the prevention of CCH and/or that showed efficacy over placebo in RCTs include verapamil, lithium, oral or iv steroids, greater occipital nerve infiltration, topiramate, methysergide, ergots, civamide and long acting triptans. Among them verapamil has better documentation [13,14]. Some agents may be not available across all European countries, others require special monitoring (e.g. verapamil, lithium, methysergide) or appropriate clinical experience (e.g. greater occipital nerve infiltration). Nevertheless patients may not respond to the above treatments. How often this happens remains unknown, but all specialists agree that a proportion of CCH patients do sometimes fail to manage their headache attacks. The urgency of the clinical situation at that point has led physicians and patients to try unusual treatments, and for most of these remedies, evidence is sparse [15]. Therefore is essential to provide specific tools for treatment escalation and scientific documentation of second line treatments, including the invasive ones.

### Preparation and evaluation

**Figure 1:**
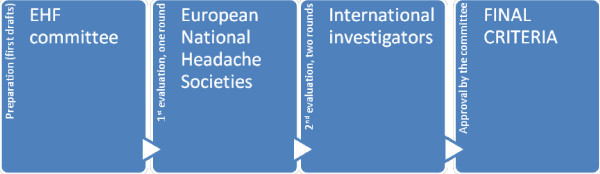
Methodology used for the preparation and evaluation of criteria.

Because of the above mentioned reasons the European Headache Federation Executive Board (EB) appointed a committee to establish diagnostic criteria for refractory CCH (rCCH). The first draft was discussed internally and approved by the EB. Two stages were followed for evaluation. In the first one the proposed criteria were sent to all representatives of the National Headache Societies of Europe for review. In a second evaluation step, all international investigators that have clinical published studies on CH were contacted for review (first and/or corresponding authors) by email. Among 85 reached 24 (28.2%) investigators listed, in the Acknowledgments section, commented and participated in a long discussion during two rounds (Figure 1), before ending the preparation of the manuscript that was then approved by all committee members (Appendix).

## Discussion

We present here (Appendix) the EHF Diagnostic Criteria for rCCH, after evaluation within EHF members of and international investigators, suggesting a specific category of TACs under the term of Cluster Headache in ICHD-3.

The current ICHD-3 beta does not include a definition of refractoriness for primary headaches. Even if a shared definition of refractoriness has already been claimed so far no consensus regarding it has emerged. The debate on what should be the key parameter for a definition of refractoriness is still open (e.g., unresponsiveness to preventive treatment, high frequency, severe disability, intolerance to current treatments, or all of these features). Considering rCCH as an evolution of CCH, we can hypothesize the inclusion of rCCH as a 3-digit diagnosis of CCH (3.1.3 Refractory chronic cluster headache). We understand that ICHD is mainly deterministic rather than phenotypic and includes only common sub-forms of primary headache disorders, but in case of rCCH the situation although rare is dramatic and requires urgent special care and management [11].

An important issue is the frequent presence of comorbidities. Depression and anxiety disorders represent undisputable co-factors in the progression of CH and require adequate treatment [16]. Other brain conditions, mainly vascular, should be carefully ruled out by appropriate investigation, including the carotid dissection [17-19]. CCH sufferers often overuse symptomatic medications to treat CH attacks and may develop medication overuse headache (MOH) in addition. In this case headaches have different phenotype than CH attacks, however, they occur primarily in patients with migrainous predisposition (ICHD-3 beta criteria) and require appropriate management [20]. Behaviours exaggerating CH attacks like alcohol consumption, smoking, taking naps, vasodilating antihypertensives should be avoided [21]. The indomethacin test is recommended to exclude paroxysmal hemicrania, since there are cases with overlapping clinical pictures [17,22]. Investigation with polysomnography may be useful to exclude several rare conditions that may exaggerate CCH [23,24]. Preventive medication should be preferably used as monotherapy but a combination is suggested when one preventive treatment is not completely effective [7]. However, care must be taken to avoid potentially negative drug interactions. There is evidence that nocebo is very prevalent among headache sufferers [25,26] and should be taken into account before treatment escalation for safety reasons.

Recently the EHF proposed new criteria for the definition of refractory chronic migraine [27]. With the same spirit, EHF members felt the need to develop new consensus criteria that define rCCH. The operational purposes of that classification are many, as RCTs involve experimental medication and neuromodulation, medical cost reimbursement, screening tool for invasive treatment or implantable devices. The new frontier for the treatment of the subset of patients with rCCH could be neuromodulation, an interesting approach but still not sufficiently validated [28].

## Conclusion

In conclusion, this consensus suggests the publication of these criteria as an Appedix in the ICHD-3 beta for a worldwide validation hopefully before their inclusion in the main body of the classification.

## Appendix

### EHF Diagnostic Criteria for refractory chronic cluster headache

Description:

Chronic cluster headache with at least three severe attacks per week despite at least three consecutive trials of adequate preventive treatments have been tested. The condition is rare, but difficult to manage and invasive treatments may be needed.

Diagnostic criteria:

A. Headache attacks fulfilling the ICHD-3 beta criteria for chronic cluster headache (CCH), or probable cluster headache (CH) and B-E criteria.

B. At least three severe CH attacks per week that impact patients’ quality of life despite preventive or symptomatic treatment.

C. Failed consecutive prophylactic treatment trials with at least three agents that showed efficacy over placebo in randomized controlled studies, used at the maximum tolerated dose over a sufficient period of time.

D. Symptomatic CCH is ruled out by negative investigation with brain MRI and MRA, eventually supplemented with carotid CT angiograms or triplex carotid ultrasound.

E. Not better accounted for by another ICHD-3 beta diagnosis.

Notes:

1. Treatments with a good clinical experience for the prevention of CCH and/or that showed efficacy over placebo in RCTs include verapamil, lithium, oral or iv steroids, greater occipital nerve infiltration, topiramate, methysergide, ergots, civamide and long acting triptans. Among them verapamil has better documentation. Some agents may be not available across all European countries.

2. Combinations of suggested preventive treatments are recommended especially when one preventive treatment decreased the attack frequency but did not controlled the situation satisfactorily, upon the physician’s decision.

3. Several preventive treatments require special monitoring (e.g. verapamil, lithium, methysergide) or appropriate clinical experience (e.g. greater occipital nerve infiltration).

Comments:

Other primary headache disorders that may mimic and/or overlap with CCH include persistent idiopathic facial pain (PIFP), SUNA, SUNCT, cluster-tic syndrome or paroxysmal hemicrania (PH); these conditions should be ruled out and/or treated for the patient to become headache-free. The indomethacin test is recommended to exclude PH. Investigation with polysomnography may be useful to exclude several rare conditions that may exaggerate CCH. CCH sufferers often overuse symptomatic medications to treat CH attacks and may develop medication overuse headache (MOH) in addition. In this case headaches have different phenotype than CH attacks, occur primarily in patients with migrainous predisposition (ICHD-3 beta criteria) and require appropriate management. When recurrent CH attacks persist despite the preventive treatment but respond to acute symptomatic, the treating physician together with the patient makes the decision for treatment escalation individually, upon the patients’ preferences and consequences in his/her personal quality of life. In this case, the situation is considered as refractory CCH as well (criterion B). Special attention to the management of simultaneous depression or other psychiatric comorbidities and nocebo behaviors is recommended.

## Competing interests

Authors declared no competing interests related to the contents of this Consensus Statement.

## Authors’ contributions

DDM designed and conceptualized the project, participated in the preparation and evaluation of the consensus and drafted and revised the manuscript for intellectual content; LE, RHJ, ZK, CH, VO, KP, AS and DV participated in the preparation and evaluation of the consensus; AN drafted the manuscript; PM conceptualized the project, participated in the preparation and evaluation of the consensus and revised the manuscript. All authors read and approved the final manuscript.
